# Reduced nuclear DNA methylation and mitochondrial transcript changes in adenomas do not associate with mtDNA methylation

**DOI:** 10.1186/s40364-018-0151-x

**Published:** 2018-12-29

**Authors:** M. J. Morris, L. B. Hesson, R. C. Poulos, R. L. Ward, J. W. H. Wong, N. A. Youngson

**Affiliations:** 1 Department of Pharmacology, School of Medical Sciences, UNSW Sydney, Sydney, NSW Australia; 2 Prince of Wales Clinical School and Lowy Cancer Research Centre, UNSW Sydney, Sydney, NSW Australia; 3 Children’s Medical Research Institute, Faculty of Medicine and Health, The University of Sydney, Westmead, NSW Australia; 4 Office of the Deputy Vice-Chancellor (Research), University of Queensland, QLD, Brisbane, Australia; 5 School of Biomedical Sciences, Li Ka Shing Faculty of Medicine, The University of Hong Kong, Pok Fu Lam, Hong Kong, Special Administrative Region of China

**Keywords:** Colorectal cancer, Adenoma, DNA methylation, mtDNA, Mitochondria

## Abstract

**Background:**

Altered mitochondrial function and large-scale changes to DNA methylation patterns in the nuclear genome are both hallmarks of colorectal cancer (CRC). Mitochondria have multiple copies of a 16 kb circular genome that contains genes that are vital for their function. While DNA methylation is known to alter the nuclear genome in CRC, it is not clear whether it could have a similar influence in mtDNA; indeed, currently, the issue of whether mitochondrial genome (mtDNA) methylation occurs is controversial. Thus our goal here was to determine whether the methylation state of mtDNA is linked to mitochondrial gene transcription in colorectal adenomas, and to assess its suitability as a biomarker in CRC.

**Methods:**

To investigate the relationship between DNA methylation and mitochondrial transcripts in adenomas, we performed RNA-sequencing and Whole Genome Bisulphite Sequencing (WGBS) of mtDNA-enriched DNA from normal mucosa and paired adenoma patient samples.

**Results:**

Transcriptional profiling indicated that adenomas had reduced mitochondrial proton transport versus normal mucosa, consistent with altered mitochondrial function. The expression of 3 tRNAs that are transcribed from mtDNA were also decreased in adenoma. Overall methylation of CG dinucleotides in the nuclear genome was reduced in adenomas (68%) compared to normal mucosa (75%, *P* < 0.01). Methylation in mtDNA was low (1%) in both normal and adenoma tissue but we observed clusters of higher methylation at the ribosomal RNA genes. Levels of methylation within these regions did not differ between normal and adenoma tissue.

**Conclusions:**

We provide evidence that low-level methylation of specific sites does exist in the mitochondrial genome but that it is not associated with mitochondrial gene transcription changes in adenomas. Furthermore, as no large scale changes to mtDNA methylation were observed it is unlikely to be a suitable biomarker for early-stage CRC.

**Electronic supplementary material:**

The online version of this article (10.1186/s40364-018-0151-x) contains supplementary material, which is available to authorized users.

## Background

The molecular development of colorectal cancer (CRC) is complex with multiple genetic mutations and epigenetic changes driving the transition from normal mucosal tissue to adenoma and ultimately invasive cancer [1]. The changes in cell biology through this transitional process are extensive and include alteration of growth and proliferation, apoptosis, motility and cell metabolism. The latter change is intimately linked to mitochondria which are the major site of energy production in the cell and the source of many substrates for anabolism [2]. A meta-analysis of proteomic studies revealed that 20% of proteins that have been repeatedly found to be differentially expressed in CRC compared to normal tissue are located in the mitochondria [3]. The importance of mitochondria in CRC has led to investigations into the potential use of mitochondrial markers for detection, staging or molecular classification of tumours, as well as in evaluating therapeutic approaches that target mitochondria [1, 4].

DNA methylation of cytosine nucleotides is the most studied and best understood epigenetic modification. It is now recognised as an important regulator of gene transcription in plants and animals [5]. In mammalian cells DNA methylation occurs primarily in the context of CG (CpG) dinucleotides, however cytosine methylation in other contexts (CHG, CHH) has been detected at high levels in neurons and stem cells [6]. The gene regulatory function of CG methylation has been largely determined through the study of genes in nuclear DNA. Mitochondria contain a separate DNA molecule (mtDNA) that is transcribed to code for proteins, ribosomal RNA and transfer RNA which are critical for mitochondrial function. Because of the importance of DNA methylation for gene regulation in the nuclear genome there has been great interest in determining whether it has similar importance in mtDNA.

However, establishing the function, or even the presence of DNA methylation in mitochondria remains controversial even after over 4 decades of research [7–9]. The difficulty in reaching a definitive conclusion likely stems from multiple factors. These include 1) when it is detected, the level of methylation is usually low, raising the possibility of it being artefactual, 2) each of the various techniques that have been used for investigation has its own inherent weaknesses, 3) there are conflicting reports on which enzyme may catalyse the methylation of mtDNA [10–12], 4) there is potentially large biological variation in the levels of mtDNA methylation due to factors such as cell/tissue-type, developmental stage, environment, and genetic background and, 5) mtDNA originates from a prokaryotic endosymbiont and differs in several fundamental aspects from the eukaryotic nuclear genome (e.g. circular rather than linear genome, DNA packaged in nucleoids rather than chromatin), therefore methodologies commonly used for investigating nuclear DNA methylation may not be suitable for analysing mtDNA methylation [13].

Some studies have reported the absence of mtDNA methylation [14, 15], however, a majority have detected it [10, 16–19]. Furthermore, there has been a recent effort to improve research in the field by recommending techniques to be used for identification of mtDNA methylation that are particularly aimed at reducing overestimation of methylation that is often artefactual [19–22].

Analysis of CRC has shown reduced mtDNA methylation in advanced tumour stage, increased mtDNA copy number and increased expression of mtDNA genes [16, 17]. Several studies have manipulated immortalised cells that were derived from colorectal tumours to investigate the causes and consequences of mtDNA methylation [10, 18, 23]. Furthermore, studies of human and rodent tissues [24] and a study in a CRC cell line [18] have added to the growing evidence that non-CG methylation may be more important than CG methylation in mtDNA. However, the extent and relevance of mtDNA methylation has not yet been confirmed. In a recent study in CRC cells, bacterially-derived CpG and virally-derived GpC methyltransferases were targeted to mitochondria [18]. Increasing CG methylation decreased mtDNA copy number, while increasing GpC methylation decreased the abundance of transcripts from mtDNA. However, more work is needed to allow an understanding of the effects of naturally-occurring mtDNA methylation in vivo.

Adenomas, as an early detectable tissue-type in the progression from normal mucosa to CRC are an attractive target lesion for prevention and intervention. Few studies have examined mitochondrial changes at the early stages of CRC. Wallace et al. (2016) reported an intriguing switch during tumour progression whereby, compared to normal tissue, mitochondrial gene expression was reduced in adenomas but increased in later stage adenocarcinomas [25]. There are conflicting reports on mtDNA copy number in adenomas compared to paired normal tissue, with increases described in one study [26] and decreases in another [27].

No previous studies have examined mtDNA methylation in adenomas and it is unclear whether abnormal mtDNA methylation is associated with gene expression changes, as seen in the nuclear genome. Furthermore, the relationship between mtDNA methylation and mtDNA copy number in adenomas, and the relative timing of mtDNA methylation changes in the transition from normal mucosa to carcinoma are also yet to be explored.

In this study we examined paired normal and adenoma tissues to determine whether mtDNA methylation changes occur in concert with mitochondrial gene expression and/or copy number changes in adenomas compared with normal tissues. We identified widespread gene expression changes indicating altered mitochondrial function but could not identify any DNA methylation changes in mtDNA in spite of a significant reduction in the nuclear genome methylation.

## Methods

### Tissue samples

DNA from CRC cell lines was obtained and extracted as previously described. For WGBS, adenomas and matched normal mucosa were obtained from 4 patients (3 males; mean age 62 years; range, 51–70 years) using endoscopic mucosal resection (consecutively collected by M.J. Bourke, between 2009 and 2011 at Westmead Hospital, Sydney; ethics approval 2009/6/4.6 and 11,194). These adenomas were mixed flat and nodular lesions, and the flat and nodular components were analysed separately. Histologic assessment indicated lesions showed no evidence of submucosal invasion. Fresh colorectal cancer tissues were obtained from surgical resection specimens at St. Vincent’s Hospital, Sydney (ethics numbers H00/022 and 00113). Patient and pathology information on clinical samples is in Additional file 1: Table S1 along with an indication of which samples were used for which experiments.

### RNA-sequencing

Next generation RNA sequencing of patient samples was performed as previously described [28, 29]. RNA sequences were aligned using TopHat and the analyzeRNA tool in the HOMER package was used to generate read counts for nuclear encoded genes while HT-seq was used to count reads for the mitochondria genes (Heinz et al., 2010) [30﻿]. These read counts were normalised using the trimmed mean of m-value method (Robinson and Oshlack, 2010) [31﻿] across samples using genes in both the nuclear genome and mtDNA (Human GRCh37/hg19 genome). A Principal Component Analysis (PCA) was generated using expression values for each identifier, with expression values generated from RNA-seq (Additional file 2: Figure S2). The same statistical analysis was performed for nuclear and mtDNA genes. The difference in the expression level of each gene between each adenoma sample and its paired normal mucosa sample was calculated. These values were used to identify statistically significant differences in adenoma compared to normal mucosa with a Student’s t-test and a Benjamini-Hochberg procedure (FDR 0.25) to control for multiple testing. Nuclear genes with an adjusted *p*-value < 0.05 that also had an average increase or decrease of 20% compared to normal mucosa were analysed with the Database for Annotation, Visualization and Integrated Discovery (DAVID). Normalised gene expression levels in the nuclear and mtDNA genome analyses for all samples are in Additional file 3.

### Whole genome Bisulphite sequencing and analysis

Five micrograms of genomic DNA from tissue samples was run on a 1% agarose gel and the 10-20 kb region excised and isolated with a Qiaex II gel extraction kit (Qiagen) to enrich for mtDNA. 5 ng of unmethylated lambda DNA (Promega) was spiked into each sample prior to conversion with an EpiTect bisufite kit (Qiagen). Libraries for next-generation sequencing were generated with a TruSeq DNA Methylation kit (Illumina) and sequenced on an Illumina HiSeq 2500 with 125 bp paired-end reads. Low quality scoring reads and adaptor sequences were removed using Trimmomatic (version 0.36). Remaining reads, 1,885,212–2,927,332 from normal mucosa, 436,044–3,244,228 from adenoma were aligned to the human genome (hg19) and methylation levels analysed using Bismark (version 0.16.1) [32]. Final read counts are in the Additional file 3. Circos plots [33] were generated to show methylated cytosines and sequence coverage for normal and adenoma mtDNA. The mappability track showing uniqueness of 35 bp Windows was from ENCODE/OpenChrom(Duke) (UCSC accession: wgEncodeEH000325). This track highlights regions of mtDNA with high homology to the nuclear genome.

For PCA of the WGBS data the percentage methylation for each sample was calculated at each nucleotide where there was at least one read recorded. Then, an average methylation value was obtained by averaging methylation at all nucleotides within 100 kb bins across the genome (a smaller bin was used at the end of each chromosome) using BEDTools [34]. A PCA was performed using data from bins where there was a methylation value recorded in all samples (Additional file 2: Figure S2).

### Bisulphite pyrosequencing

This technique determines the relative proportions of cytosine (indicating a methylated cytosine) and thymine (indicating an unmethylated, and consequently bisulphite converted cytosine) nucleotides at individual cytosine sites in PCR product from regions of interest. The patient samples used overlapped with the WGBS and RNA-seq analyses and are indicated in Additional file 1: Table S1. 300 ng of patient sample genomic DNA was digested with *SphI* restriction endonuclease (New England Biolabs) for 10 mins at 37 °C then incubated at 65 °C for 20 mins to inactivate the enzyme. The samples were then bisulphite converted with an EpiTect bisufite kit (Qiagen) using the manufacturer’s Protocol for Low Concentration Solutions. The *SphI* digestion linearises the mtDNA which has recently been shown to be required for effective bisulphite conversion [19, 21]. The MT-RNR2 and MT-CO1 regions were amplified from bisulphite-converted DNA with MyTaq HS DNA polymerase (Bioline) using primers from Liu et al., 2016 [19], (named primer sets MT5 and MT12 in that paper). Pyrosequencing was performed on a Q24 Pyrosequencer (Qiagen) with PyroMark Gold reagents (Qiagen). Data from normal mucosa and adenoma groups were compared by using Student’s t-test.

### mtDNA copy number qPCR

Mitochondrial DNA copy number was measured (prior to mtDNA enrichment) by qPCR on a LightCycler480 (Roche). Representative regions of the mtDNA (16S ribosomal RNA gene) and the nuclear genome (Nestin gene) were amplified and relative levels compared with the delta delta Ct method. An aliquot was taken from all samples was used to calibrate the delta delta Ct calculation. Primers used were; 16S F: CGAAAGGACAAGAGAAATAAGG; 16S R: CTGTAAAGTTTTAAGTTTTATGCC; Nestin F: AAACCAGAGCCATGAGACAC; Nestin R: TGGCCTACAGCCTCTTTTTC. For analysis of patient samples, the mtDNA copy number from each normal sample was given the value 100% and the relative change in the paired adenoma sample was calculated. Data from normal mucosa and adenoma groups were compared by using Student’s t-test.

## Results

### Transcriptome analysis suggests that mitochondrial energy production is reduced in adenomas

To find differentially regulated pathways in normal and adenoma tissues, we analysed RNA-seq data from 6 normal mucosal and 12 matched adenoma samples and identified 1846 and 1230 genes that were significantly downregulated and upregulated, respectively in adenomas (*P* < 0.05 Multiple testing error was controlled with the Benjamini-Hochberg Procedure, the critical value was calculated with a false discovery rate of 25%.). Analysis of these genes with the Database for Annotation, Visualization and Integrated Discovery (DAVID) found 1 Biological Processes that was significantly downregulated in adenomas after Benjamini correction (P < 0.05). This was ATP hydrolysis coupled proton transport which consists of genes required for establishment of the proton gradient that drives ATP production in the mitochondria (Table 1). Other mitochondrial-associated Biological Processes were significantly enriched in the downregulated genes but did not pass Benjamini Correction. These included Ion transmembrane transport and the Tricarboxylic acid cycle (Table 1). Overall these data suggest that the adenomas have reduced mitochondrial energy production compared to normal mucosa. Twenty-one Biological Processes were significantly upregulated in adenoma samples after Benjamini correction. Many of these processes were indicative of the increases in cellular substrate biogenesis required for the increased cell division of the transformation process (Table 1). Therefore our transcriptome analyses suggest that mitochondrial energy production is reduced, and generation of substrates for cell division is increased in adenomas.

**Table 1:** Pathway enrichment for differentially expressed genes in adenoma vs normal mucosa

Enriched Gene Ontology Biological Process category	Number of genes altered in pathway	*P* value	Benjamini corrected enrichment (*P*)
Downregulated in adenoma			
ATP hydrolysis coupled proton transport	13	1.50E-05	3.50E-02
Ion transmembrane transport	39	4.90E-05	5.40E-02
Tricarboxylic acid cycle	9	4.00E-03	4.40E-01
Upregulated in adenoma			
Translational initiation	54	2.20E-28	7.80E-25
rRNA processing	67	2.80E-28	4.80E-25
Transcription, DNA-templated	204	3.60E-13	1.80E-10
Ribosome biogenesis	10	3.30E-04	5.00E-02
Multicellular organism growth	15	4.80E-04	6.70E-02

### A subset of mtDNA-encoded genes are reduced in adenomas

**Fig. 1:**
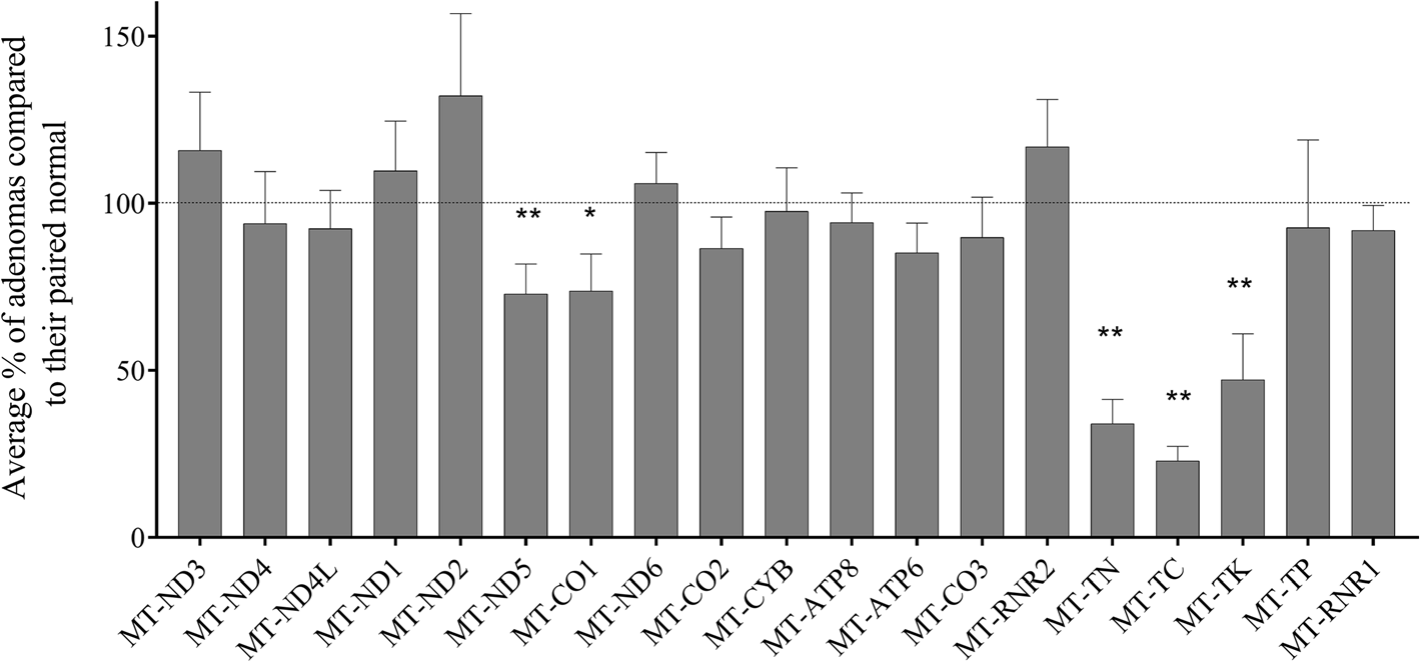
Comparison of mtDNA-transcript levels in 8 adenomas compared to 4 matched normal mucosal samples revealed that transcript levels of 2 protein coding genes and 3 tRNAs are reduced in adenomas. 100% means no change between normal and paired adenoma. Average of 8 comparisons +/-SEM shown. Only tRNAs that had sequence reads are displayed. *P < 0.05, **P < 0.009 normal vs adenoma. After Benjamini-Hochberg correction, MT-CO1 and MT-ND5 were just outside the critical value (Q = 0.053, and 0.066 respectively). MT-ND1–6, NADH dehydrogenase subunits 1–6; MT-CO1–3, Cytochrome c oxidase subunits I-III; MT-CYB, Cytochrome b; MT-ATP6, ATP synthase Fo subunit 6; MT-ATP8, ATP synthase protein 8; MT-RNR2, mitochondrial ribosomal 16S RNA; MT-RNR1, MT-TN, tRNA asparagine; MT-TC, tRNA cysteine; MT-TK, tRNA lysine; MT-TP tRNA proline; mitochondrial ribosomal 12S RNA

The initial RNA-seq analysis pipeline did not quantify genes that are transcribed from the mtDNA. Therefore a separate analysis of 4 normal mucosa and 8 matched adenoma samples (2 from each patient) was performed. It revealed that genes MT-CO1, MT-ND5 and three tRNAs (asparagine, cysteine and lysine) were reduced in adenomas (Fig. 1). After application of the Benjamini-Hochberg False Discovery Rate Procedure, MT-CO1 and MT-ND5 were just outside the Benjamini-Hochberg critical value (Q = 0.053, and 0.066 respectively).

### Whole genome Bisulphite sequencing (WGBS) revealed regional differences in mtDNA methylation

To identify regions of mtDNA that contain methylation we performed whole genome bisulphite sequencing (WGBS) on DNA from 4 normal mucosa and adenoma paired samples, plus an additional normal mucosa from a patient with CRC. A summary of the percentage of cytosines in CG, CHG and CHH contexts that resisted sodium bisulphite conversion in the nuclear genome, mtDNA and lambda control DNA is presented in Table 2. In nuclear DNA, the level of CG methylation was significantly lower in adenoma than normal mucosa (*P* < 0.01). In contrast no large scale differences in methylation level were seen in mtDNA. In all samples the level of mtDNA CG methylation (~ 1%) was much lower than in the nuclear genome (~ 70%). Non-CG methylation was also consistently ~ 1% in all groups. As the spiked lambda DNA controls revealed that ~ 99% of cytosines were converted by bisulphite treatment it is not possible to determine if the WGBS data reflect significant group differences (between normal and adenoma) in mtDNA, or are caused by slight differences in the effectiveness of bisulphite conversion between groups.

**Table 2:** Methylation summary of whole genome bisulphite sequencing

	Normal mucosa (*n* = 5)	Adenoma (*n* = 4)
Nuclear Genome		
CG methylation %	74.5 ± 0.50	67.9 ± 1.16*
CHG methylation %	1.3 ± 0.06	1.0 ± 0.05
CHH methylation %	1.3 ± 0.07	1.0 ± 0.05
Mitochondrial Genome		
CG methylation %	1.0 ± 0.09	1.0 ± 0.16
CHG methylation %	1.3 ± 0.14	0.8 ± 0.14
CHH methylation %	1.2 ± 0.06	0.92 ± 0.12
Lambda control % unconverted cytosines	1.06 ± 0.06	0.86 ± 0.05

Average methylation percentage ± SEM, *P < 0.01 Normal vs Adenoma. No statistical tests were performed on methylation levels within the range of the lambda controls (which indicate conversion efficiency)

**Fig. 2:**
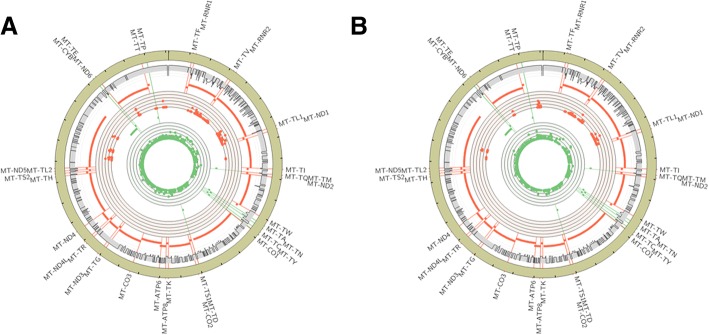
Circos plot [33] showing the locations of methylated cytosines in mtDNA from 5 normal colon mucosa (**a**) and 4 adenoma (**b**) samples. Inner scatter plot tracks indicate methylation for all sites with > = 5 total reads per locus (axis ranges from 0 to 50%; axis gridlines indicate increments of 10%) and all genes for the minus/light strand (green dots), and plus/heavy strand (red dots). The outer (grey) track indicates uniqueness of 35 bp windows, with axis lines indicating increments of 0.25, from 0 (less unique; inner) to 1 (more unique; outer) where axis values between 0.5 and 1 are shaded in grey. The transcription start site of each gene is indicated with a radial line, and the name of each gene is listed around the outside of the chromosome. 500 nucleotide increments within the chromosome (beige outer track) are indicated with tick marks

Methylated cytosines were mapped on the heavy and light strands of mtDNA (Fig. 2). While the average methylation level was uniformly low on both strands (~ 1%) frequent methylation of specific cytosines was detected. On the minus/light strand, clusters of highly (~ 20%) methylated cytosines were observed at the highly transcribed ribosomal RNA genes. However, when the 12S and 16S rRNA gene regions was examined further, no significant differences were found between normal mucosa and adenoma in the frequency of methylated cytosines.

There was a large difference in the number of sequence reads that originated from the different strands. There were ~10x more reads from the minus/light strand than from the plus/heavy strand. This has been reported before in bisulphite-treated DNA sequencing and is thought to be due to DNA degradation when the depurination step of the process causes random strand breaks [35]. It is possible that the considerable differences in cytosines in the heavy and light strands (5193 in plus strand, 2181 in minus strands in hg19) led to differential levels of degradation during the sodium bisulphite treatments.

To validate the WGBS result we performed locus-specific pyrosequencing of cytosines located at the 16S rRNA (MT-RNR2 gene) and the MT-CO1 gene (proximal to the differentially expressed tRNA genes MT-TN, MT-TC). Consistent with the WGBS the pyrosequencing detected low methylation levels in mtDNA in both normal mucosa and adenoma (Additional file 4: Figure S1). Furthermore, no significant difference in methylation level between normal mucosa and adenoma was identified in the region of MT-CO1/MT-TC/MT-TN although 1 CpG in MT-RNR1 did have a small increase (3.0% in normal mucosa and 4.5% in adenoma, *p* = 0.007).

Examination of mtDNA copy number detected no difference between paired normal/adenoma patient samples (adenomas were on average 103 ± 31% the level of their paired normal sample).

## Discussion

By investigating mtDNA methylation in adenomas we aimed to increase the knowledge on the molecular events in this early-stage of carcinogenesis and to evaluate the likelihood that it could be used as a biomarker for CRC risk. Methylation of a nuclear gene that encodes a mitochondrial protein (UQCRB) has been identified as a potential biomarker in colorectal cancers [36]. Furthermore, mtDNA methylation has also been identified as a biomarker for a range of conditions [13] such as air pollution exposure during pregnancy [37], nonalcoholic steatohepatitis (NASH) [38] and inherited risk for breast cancer [39]. However, while there are studies that have investigated mtDNA methylation in carcinomas, i.e. cells that are late in the adenoma-carcinoma sequence [16, 17], no previous studies have examined mtDNA methylation in the early-stage cell type, adenomas.

In patient samples we observed transcription changes which suggest that mitochondrial function was impaired in adenomas compared to normal mucosa. This fits with the shift in cancer cells from oxidative metabolism, to glycolysis for energy production [40]. A recent study examined this process in detail in CRC and detected transcriptome and metabolome changes (including reductions in fatty acid catabolism and the TCA cycle) in the adenoma stage of the adenoma–carcinoma sequence [41]. In contrast to the early changes in mitochondrial transcription, our data suggest that mtDNA methylation changes are not a feature of adenomas. There is variability in reports of mtDNA copy number changes in adenomas [26, 27]. A potential explanation is that copy number differences reflect the diversity of colorectal lesions that are labelled with the term adenoma and the variable level of dysplasia possible between different adenomas.

Our observation of reduced genome-wide CpG methylation in adenoma may fit with a previous study that identified reduced LINE-1 methylation in adenomas (particularly in patients with adenomas and concomitant CRC) [42]. LINE-1 elements are the most abundant class of retrotransposon in the human genome and are often used as a proxy indicator for whole genome methylation. Interestingly, higher genome-wide methylation (as measured by a sensitive HPLC methodology) in circulating leukocytes was found to associate with reduced risk for colorectal adenomas in women [43]. It is unclear whether the determinants of leukocyte nuclear DNA methylation reduction are the same as those that lead to its reduction adenomas. However, future studies that compare the two tissues may reveal mechanistic links of importance for CRC risk. Two studies from the same team have examined mtDNA methylation in CRC. Both studies correlated reduced D-loop methylation in CRC with increased expression of a mtDNA-encoded gene (ND2) [16, 17], and one also correlated it with increased mtDNA copy number [17]. Therefore, reductions in mtDNA methylation may be a feature of later stage CRC. However, whether it is a cause or consequence of the mitochondrial functional changes remains to be determined.

Similar to previous reports, our WGBS data showed that mtDNA has a much lower level of CpG methylation than the nuclear genome [44, 45]. We also found non-CG methylation in mtDNA, but at a similarly low level as the nuclear genome. As mentioned above it was not possible to statistically compare the methylation levels across the whole mtDNA as the ~ 1% methylation is within the range of the lambda DNA controls which indicated that ~ 1% of the cytosines could have escaped sodium bisulphite conversion. Additionally, there is still a possibility that the methylation level is overestimated or artefactual due to secondary structures of the mtDNA inhibiting bisulphite conversion. We did not linearise the mtDNA prior to bisulphite conversion for WGBS as has been recently recommended as the best practice [20, 22], however, we did do this in the subsequent pyrosequencing experiments. Nonetheless our data do suggest that there is no large gain of methylation in adenomas, which is of value for evaluating its use as a biomarker in early-stage CRC. It should be noted that the low sample-size in the WGBS and pyrosequencing experiments, makes it important that further studies of adenoma mtDNA methylation are required to confirm our findings.

Multiple studies have described regional differences in the level of methylation across the 16Kb of human mtDNA [10, 12, 19, 20, 44]. These studies found the highest levels of methylation at the D-loop [19], ND2, ND4–6, and CYB genes [44], or the rRNA [10, 12, 19, 20] and ATP6 genes [10]. We found the highest level of methylation to be in the rRNA genes which is in agreement with some of the studies with the highest technical rigour [19, 20]. We compared these gene regions in the normal and adenoma samples as the methylation levels were above the ~ 1% background of unconverted cytosines. However, no significant group differences were observed, and no transcriptional differences in the highly transcribed 12S (MT-RNR1) and 16S (MT-RNR2) rRNA genes was seen in adenomas (Fig. 1), so it is unclear whether this methylation is functional.

Our observation of increased methylation at the rRNA gene regions could be influenced by them containing relatively high levels of 5-hydroxymethylcytosine (5hmC) as well as 5-methylcytosine [10]. As sodium bisulphite sequencing cannot distinguish between 5hmC and 5mC [46] the high levels that we detected may represent the sum of these two base variants. This explanation could be investigated with either antibody or oxidative bisulphite [47] methods that can distinguish 5mC and 5hmC. Alternatively, the differences in regional mtDNA methylation patterns may be due to the methylation/demethylation being dynamic and influenced by functional changes in mtDNA transcription, replication or mitochondrial activity.

A final potential explanation for the regional differences in mtDNA methylation and coverage could be copy number changes within the mtDNA molecule. Rare cases have been reported of mtDNA deletions and rearrangements in patients with mitochondrial disease [48] and neuromuscular disease [49]. The regional pattern of methylation in mtDNA that we observed was similar between normal and disease tissues which could support the conclusion that the patterns are not caused by (pathology-associated) copy number changes. However, future long-read sequencing of mtDNA in mucosa and CRC is needed to confirm this.

## Conclusions

In conclusion, our study suggests that while alteration to mitochondrial function is a feature of all adenomas, extensive alteration of mtDNA methylation is not. Therefore mtDNA methylation is unlikely to be suitable as an early biomarker for CRC.

## Additional files

Additional file 1: **Table S1.** Clinical sample and patient information and experimental usage. (DOCX 15 kb)

Additional file 2: **Figure S2.** Quality control of ‘omics’. A Volcano plot depicting fold changes associated with differentially expressed nuclear genes in paired normal mucosa vs adenoma samples (*n* = 6 v12 respectively). B Principal Component Analysis of RNA-sequencing (6 normal mucosa, 12 adenoma samples) using expression values for each identifier. C Principal Component Analysis of Whole Genome Bisulphite Sequencing (5 normal mucosa, 4 adenoma samples) using average methylation in 100 kb bins, for regions where all 9 samples had at least one read. (DOCX 4237 kb)

Additional file 3: Supplementary data. Nuclear and mtDNA RNA-seq gene expression levels. WGBS read counts. (XLSX 10324 kb)

Additional file 4: **Figure S1.** DNA methylation levels at 4 CpG sites in MT-RNR1 and 2 CpG sites in MT-CO1 detected with pyrosequencing in normal mucosa (*n* = 3) and adenoma (*n* = 4) patient samples. Mean ± SEM displayed. N, normal mucosa; A, adenoma. No error bars indicates identical measurement in samples. (DOCX 57 kb)

## Abbreviations

5hmC: 5-hydroxymethylcytosine
5mC: 5-methylcytosine
CRC: Colorectal cancer
DAVID: Database for Annotation, Visualization and Integrated Discovery
HPLC: High Performace Liquid Chromatography
LINE-1: Long Interspersed Nuclear Element-1
mtDNA: Mitochondrial DNA
NASH: Non-alcoholic steatohepatitis
PCA: Principal component analysis
rRNA: Ribosomal RNA
TCA: Tricarboxylic acid
WGBS: Whole Genome Bisulphite Sequencing
