# Supplementary material for: Reduced nuclear DNA methylation and mitochondrial transcript changes in adenomas do not associate with mtDNA methylation
Source: Biomark Res. 2018 Dec 29;6:37. doi: 10.1186/s40364-018-0151-x (PMC6311003; doi:10.1186/s40364-018-0151-x)
Supplement: Supplementary file 1 — Table S1. Clinical sample and patient information and experimental usage. (DOCX 15 kb) [file 40364_2018_151_MOESM1_ESM.docx]

**Table S1**. Clinical sample and patient information and experimental usage

| Lesion ID | Paris Classification | Age | Sex | Size (mm) | Site in Large Bowel | Morphology | Dysplasia | Histopathological type | RNA-seq nuclear genes (N), RNA-seq mtDNA genes (M), WGBS (W), mtDNA copy number PCR (C), Prosequencing (P) |
| --- | --- | --- | --- | --- | --- | --- | --- | --- | --- |
| ISTA 1 | IIa+Is | 78 | F | 60 | Caecum | Granular | LGD | Tubulovillous adenoma | N, M, C, P |
| ISTA 2 | IIa+Is | 65 | M | 100 | Rectum | Granular | LGD | Tubulovillous adenoma | N, M, W, C, P |
| ISTA 3 | IIa+Is | 62 | M | 40 | Rectum | Granular | LGD | Tubulovillous adenoma | N, M, W, C, P |
| ISTA 4 | IIa+Is | 51 | F | 30 | Hepatic flexure | Granular | LGD | Tubulovillous adenoma | N, W, C, P |
| ISTA 5 | IIa+Is | 87 | M | 35 | Caecum | Granular | LGD | Tubulovillous adenoma | N |
| NG | IIa |  |  | 25 | Ascending colon | Non-granular | LGD | Tubular adenoma | N, M, C |
| 82 | IIa | 70 | M | 35 | Transverse colon | Non-granular | LGD | Tubular adenoma | W |
| 85 | IIa | 75 | M | 40 | Transverse colon | Non-granular | LGD | Tubular adenoma | C |
| 25912/4235 |  | 23 | M |  | Bowel, - colon, sigmoid |  |  | Carcinoma | W (on matched normal mucosa) |
