# Supplementary material for: Reduced nuclear DNA methylation and mitochondrial transcript changes in adenomas do not associate with mtDNA methylation
Source: Biomark Res. 2018 Dec 29;6:37. doi: 10.1186/s40364-018-0151-x (PMC6311003; doi:10.1186/s40364-018-0151-x)
Supplement: Supplementary file 2 — Figure S2. Quality control of ‘omics’. A Volcano plot depicting fold changes associated with differentially expressed nuclear genes in paired normal mucosa vs adenoma samples (n = 6 v12 respectively). B Principal Component Analysis of RNA-sequencing (6 normal mucosa, 12 adenoma samples) using expression values for each identifier. C Principal Component Analysis of Whole Genome Bisulphite Sequencing (5 normal mucosa, 4 adenoma samples) using average methylation in 100 kb bins, for regions where all 9 samples had at least one read. (DOCX 4237 kb) [file 40364_2018_151_MOESM2_ESM.docx]

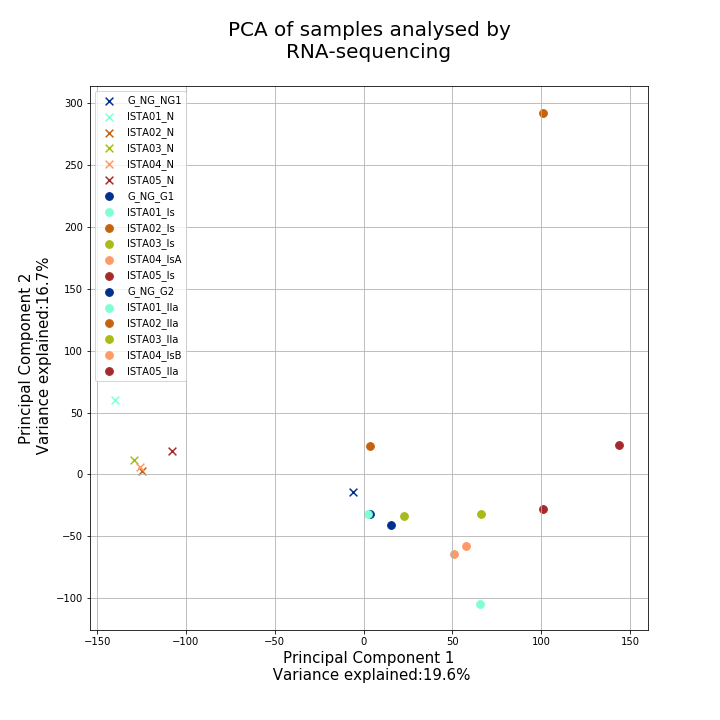


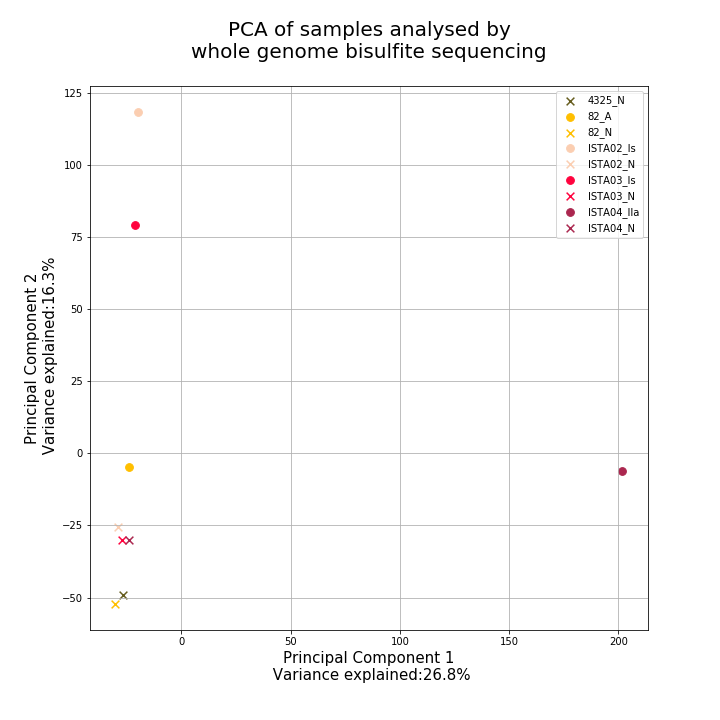


Figure S2. Quality control of ‘omics’

1. Volcano plot depicting fold changes associated with differentially expressed nuclear genes in paired normal mucosa vs adenoma samples (n=6 v12 respectively).
2. Principal Component Analysis of RNA-sequencing (6 normal mucosa, 12 adenoma samples) using expression values for each identifier.
3. Principal Component Analysis of Whole Genome Bisulphite Sequencing (5 normal mucosa, 4 adenoma samples) using average methylation in 100kb bins, for regions where all 9 samples had at least one read.
