# Supplementary material for: Reduced nuclear DNA methylation and mitochondrial transcript changes in adenomas do not associate with mtDNA methylation
Source: Biomark Res. 2018 Dec 29;6:37. doi: 10.1186/s40364-018-0151-x (PMC6311003; doi:10.1186/s40364-018-0151-x)
Supplement: Supplementary file 4 — Figure S1. DNA methylation levels at 4 CpG sites in MT-RNR1 and 2 CpG sites in MT-CO1 detected with pyrosequencing in normal mucosa (n = 3) and adenoma (n = 4) patient samples. Mean ± SEM displayed. N, normal mucosa; A, adenoma. No error bars indicates identical measurement in samples. (DOCX 57 kb) [file 40364_2018_151_MOESM4_ESM.docx]

Figure S1. DNA methylation levels at 4 CpG sites in MT-RNR1 and 2 CpG sites in MT-CO1 detected with pyrosequencing in normal mucosa (n=3) and adenoma (n=4) patient samples. Mean ± SEM displayed. N, normal mucosa; A, adenoma. No error bars indicates identical measurement in samples.
